# Supplementary figures and images for: Transformation of cereal grains: Botanical and chemical analysis of food residues encrusted on pottery from the Funnel Beaker settlement of Oldenburg LA 77, northern Germany
Source: PLoS One. 2024 Jan 19;19(1):e0296986. doi: 10.1371/journal.pone.0296986 (PMC10798637; doi:10.1371/journal.pone.0296986)

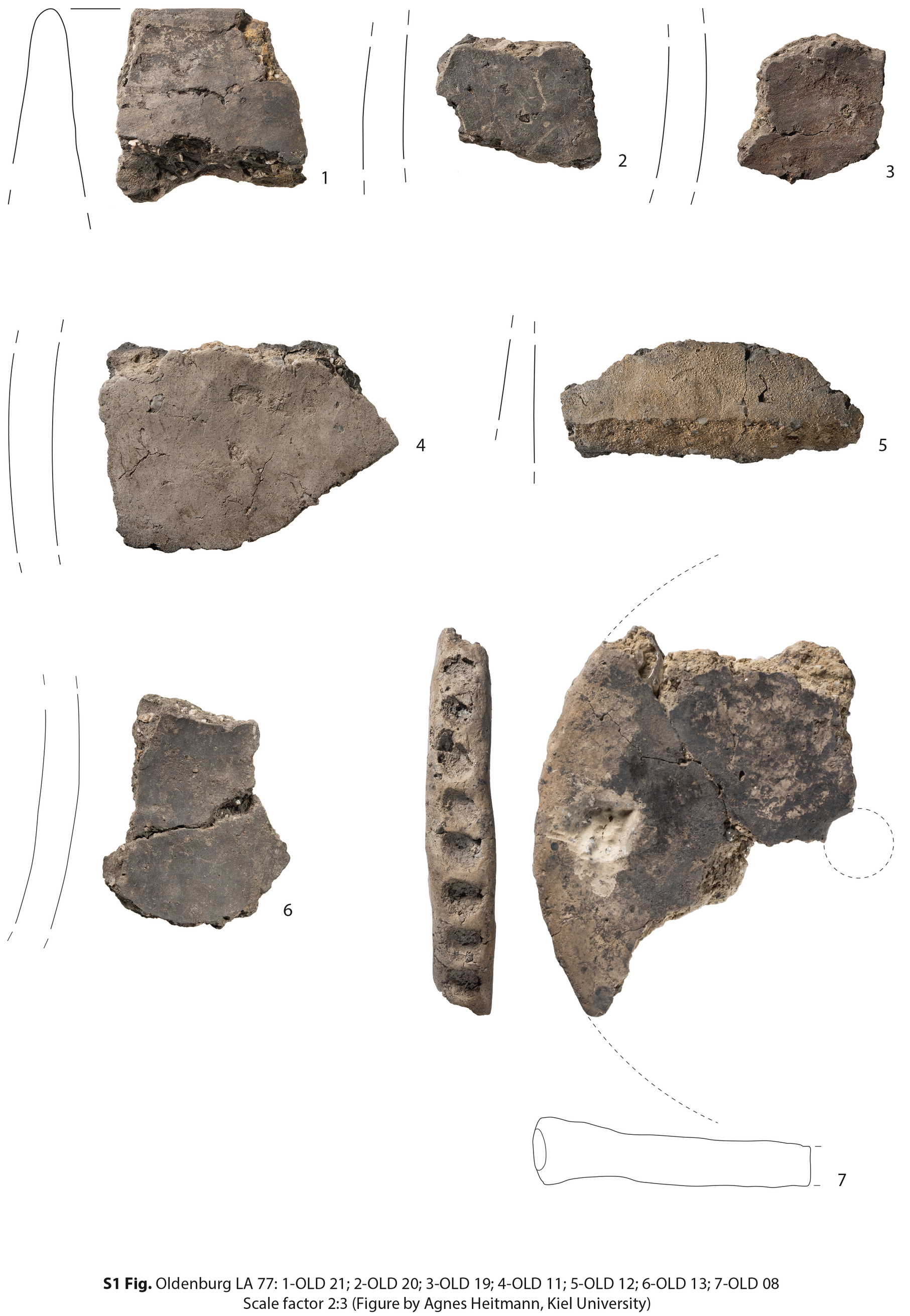

Supplement: S1 Fig — (TIF) [file pone.0296986.s001.tif]

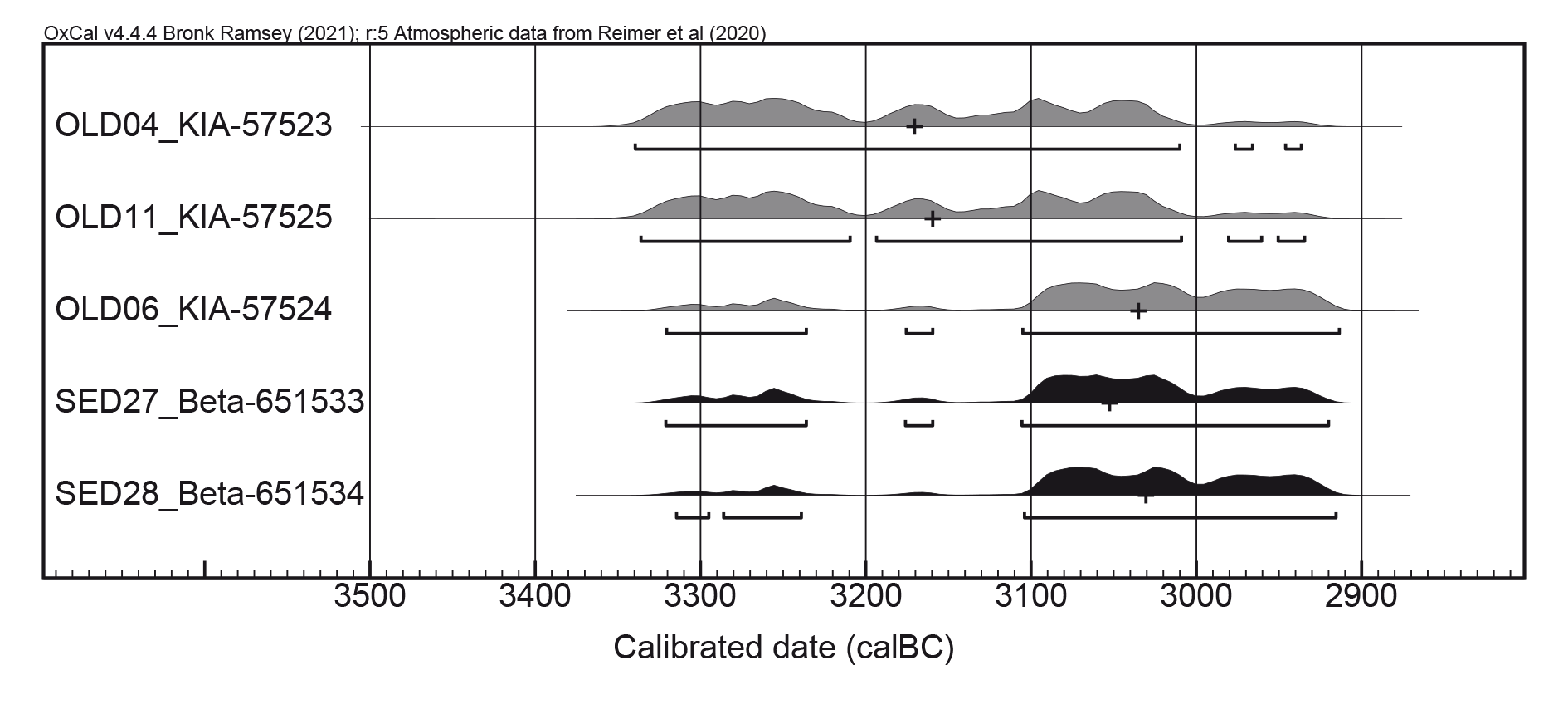

Supplement: S2 Fig — Filipović). (TIF) [file pone.0296986.s002.tif]
