# Supplementary material for: Transformation of cereal grains: Botanical and chemical analysis of food residues encrusted on pottery from the Funnel Beaker settlement of Oldenburg LA 77, northern Germany
Source: PLoS One. 2024 Jan 19;19(1):e0296986. doi: 10.1371/journal.pone.0296986 (PMC10798637; doi:10.1371/journal.pone.0296986)
Supplement: S1 Table — Filipović). (DOCX) [file pone.0296986.s003.docx]

| **Sample** | **Material** | **Laboratory code** | **Radiocarbon age** | **Unmodelled (BC/AD)** | | | |
| --- | --- | --- | --- | --- | --- | --- | --- |
|  |  |  |  | **from** | **to** | **%** | **median** |
| OLD 04 | foodcrust | KIA-57523 | 4455 ± 35 BP | -3340 | -2937 | 95.4 | -3171 |
| OLD 11 | foodcrust | KIA-57525 | 4450 ± 35 BP | -3337 | -2935 | 95.4 | -3160 |
| OLD 06 | foodcrust | KIA-57524 | 4410 ± 35 BP | -3321 | -2914 | 95.4 | -3036 |
| SED27 | emmer grain | Beta-651533 | 4420 ± 30 BP | -3322 | -2921 | 95.4 | -3053 |
| SED28 | emmer grain | Beta-651534 | 4410 ± 30 BP | -3315 | -2916 | 95.4 | -3031 |

**S2 Table 1.** Radiocarbon AMS-dates and their calibrated values for three samples of food crusts (OLD 04, OLD 06 and OLD 11) and two single charred emmer grains (SED27–28).
