# Supplementary material for: Transformation of cereal grains: Botanical and chemical analysis of food residues encrusted on pottery from the Funnel Beaker settlement of Oldenburg LA 77, northern Germany
Source: PLoS One. 2024 Jan 19;19(1):e0296986. doi: 10.1371/journal.pone.0296986 (PMC10798637; doi:10.1371/journal.pone.0296986)
Supplement: S4 Table — The relative intensity of the FTIR transmission bands is indicated in five categories (-: absent, +/-: minimal, +: average, ++: strong and +++: very strong). The origin of the bands is indicated as Mi = minerals; PS = polysaccharides; Pr = proteins; Al = aliphatic hydrocarbons; Ar = aromatic compounds; Lig = lignins; S = sulfates and Si = silicates. The type of transmission band is given as: s. = stretch; b. = bend; vibr. = vibration [such as rocking, scissoring, wagging, or twisting]; def. = deformation; skel. = skeletal; asym. = asymmetrical. (DOCX) [file pone.0296986.s006.docx]

**S4 Table.** ATR–FTIR results. The relative intensity of various FTIR transmission bands is indicated in five categories (-: absent, +/-: minimal, +: average, ++: strong and +++: very strong). The marker value of the bands is also indicated (Mi = minerals, PS = polysaccharides, Pr = proteins, Al = aliphatic hydrocarbons; Ar = aromatic compounds; Lig = lignins, S = sulfates, Si = silicates). trans. = transmission; s. = stretch; b. = bend; vibr. = vibration [such as rocking, scissoring, wagging, or twisting]; def. = deformation; skel. = skeletal; asym. = asymmetrical.

|  | Sample /  Transmission bands | [cm^-1^] | OLD  01  p1  in | OLD  02  p3  ex | OLD  03  p3  ex | OLD  04  p2  in | OLD  05  p1  ex | OLD  06  p1  in | OLD  07  p3  in | OLD  08  p1  top | OLD  09  p1  in | OLD  10  p1  in | OLD  11  p1  in | OLD  12  p3  in | OLD  13  p1 | OLD  14  p1 | OLD  15  p1  top | OLD  16  p2 | OLD  17  p1 | OLD 18  p1 | OLD19  p3 | OLD20  p1 | OLD  21  p1 | Wheat |
| --- | --- | --- | --- | --- | --- | --- | --- | --- | --- | --- | --- | --- | --- | --- | --- | --- | --- | --- | --- | --- | --- | --- | --- | --- |
| S | O-H (s.) | 3526 | + | − | ++ | − | − | − | +/− | ++ | +/− | − | +/− | − | − | + | +++ | +/− | + | + | +/− | +/− | + | − |
|  | O-H (s.) & N-H (s.) | 3600–3200 | +++ | +++ | +++ | +++ | ++ | +++ | ++ | ++ | +++ | +++ | +++ | +++ | +++ | +++ | − | +++ | ++ | ++ | +++ | +++ | ++ | +++ |
| S | O-H in sulfates | 3402 | ++ | +/− | ++ | − | +/− | − | +/− | ++ | + | − | + | − | + | ++ | +++ | + | + | ++ | + | +/− | + | − |
| S |  | 3242 | +/− | +/− | + | +/− | +/− | − | +/− | + | + | − | +/− | − | +/− | +/− | ++ | +/− | + | +/− | +/− | +/− | ++ | − |
| Al | C-H (s.) in -CH3 | 2952/2870 | +/− | − | − | + | − | +/− | + | +/− | +/− | +/− | +/− | − | − | − | − | +/− | +/− | +/− | +/− | +/− | − | +/− |
| Al | C-H (s.) in -CH2- | 2930/2850 | +/− | ++ | − | ++ | − | ++ | ++ | + | + | ++ | ++ | + | + | +++ | − | ++ | +/− | + | + | + | + | ++ |
| Ps | C=O(OH) (s.) | 1730 ± 10 | − | − | − | − | − | − | − | − | − | − | − | − | − | − | − | − | − | − | − | − | − | +/− |
| Ps | C=O (s.) | 1715–1695 | − | + | − | +/− | − | +/− | − | − | ++ | +/− | − | − | + | ++ | − | ++ | − | − | + | − | − | − |
| S | Sulfates (s.) | 1684 | +/− | − | +/− | − | +/− | − | − | + | − | − | + | − | − | − | + | − | − | − | − | − | + | − |
| Pr | Amide I | 1650 | − | − | − | − | − | − | − | − | +/− | − | − | − | − | − | − | − | − | − | − | +/− | − | ++ |
| Ar | C=C (s.) | 1640–1620 | − | − | − | − | − | − | − | − | − | ++ | − | − | − | − | − | − | − | − | − | ++ | − | − |
| S | Sulfates (s.) | 1620 | ++ | +++ | ? | ? | ? | ? | ++ | ++ | ++ |  | +++ | +++ | +++ | +++ | ++ | ++ | ++ | ++ | − |  | ++ | − |
| Lig | C=C in lignins | 1615–1600 | − | − | − | − | +++ | − | − | − | − | − | − | − |  |  | − | − | − | − | +++ |  | − |  |
| Al | [O-C=O]- (s.) salts | 1580 ± 30 | − | − | ? | − | − | − | +/− |  |  |  |  | +++ |  |  | − | − | − | − | − |  | +/− | − |
| Pr | Amide II | 1550 | − | − | − | − | − | − | − | − | − | − | − | − | − | − | − | − | − | − | − | +/− | − | + |
|  | C-H (def.) | 1522 | − | − | − | − | − | − |  |  |  |  |  | − |  |  | − | − | + | − | − |  | − |  |
| Al | C-H (b.) in -CH2 | 1485–1445 | − | + | − | + | − | − | − | + | − |  |  | − |  | + | − | + | + | +/− | + |  | − |  |
| PS | C=C (skel. vibr.) aromatic sugars | 1450 | − | − | − | − | − | − | − | − | − |  |  | − | − | − | − | − | − | − | − |  | − | + |
|  |  | 1435 | − | − | − | − | − | ++ | − | − | − | − | − | − | − | − | − | − | − |  | − | ++ | − |  |
| Ar | C=C (skel. vibr.) | 1425–1415 | + | − | + | − | + | − | − | − | + | ++ | ++ | − | − | − | − | − | − | − | + |  | − |  |
| PS | C-H2 (b.) in cellulose | 1410 |  | − | − | + | − | − | − | − | − |  |  | − | − | − | − | − | − | − | − |  | − | + |
| Al | [O-C=O]- (s.) salts | 1400–1380 | − | − | − | − | − |  | ++ | − | − | + | − | ++ | − | − | − | − | − | − | − | − | − | − |
| Al | C-H (b.) in -CH3 | 1385–1380 | − | + | − | − | − | + |  | + | +/− | +/− | +/− | − | + | + | − | + | + | − | − | + | − |  |
| Al | C-H (b.) in -CH3 | 1370–1365 | − | − | − | − | − | − |  | − |  | +/− |  | − | − |  | − | − | − | − | − |  | − | + |
| Ar | C-H (def. vibr.) aromatic | 1275, 1235, 1218 | − | − | − | − | − | − |  |  |  |  |  | − | + | + | − | − |  |  | − |  |  |  |
|  | C-O (s.) in pyranose ring | 1207–1200 | − | − | − | − | − | − | − | − | − | − | − | − | − | − | − | − | − | − | − | − | − | + |
|  | C-O-C (asym. s.) | 1155–1140 | − | +++ | +++ | +++ | +++ | ++ |  |  |  |  |  |  |  |  |  |  |  |  | ++ |  |  | + |
| S | S=O (s.) | 1120–1107 | + | +/− | + | +/− | +/− | − | − | − | − | − | + | +/− | +/− | +/− | +++ | +/− | + | + | +/− | +/− | +/− | − |
| Ps | C-O-C (skel. vibr.) in intact pyranose | 1080–1078 | + | + | +/− | + | + | +/− | ++ | ++ | + | +/− | +/− | +/− | − | − | − | − | − | − | +/− | + | − | ++ |
| Ps | C-O (s.) | 1043 | +/− | +/− | ++ | ++ | + | + | ++ |  |  |  |  |  |  |  |  |  |  | +++ | + |  | +++ | + |
| Ps | C-O (skel. vibr.) C-6 ring | 1030–1016. | +++ | − | − | − | − | − | − | − | ++ | − | − | − | − | − | − | − | − | − | − | − | − | ++ |
| S | S-O (b.) | 1005 | +/− | ? | ? | ? | ? | ? |  |  | +/− |  |  |  |  |  |  |  |  |  |  |  |  |  |
| Ps | C-O-C (ring vibr.) | 929/860/763 | +/− | − | + | + | − | + | − | − | − | +/− | +/− | + | + | + | − | − | − | − | + | − | − | ++ |
| Lig | Epoxy group | 823 | − | − | − | − | − | − | − | − | − | − | − | − | − | − | − | − | − | − | − | − | − | − |
| Si | Si-O in quartz | 796 & 779 | + | +/− | + | − | +/− | − | + | + | − | − | +/− | − | − | − | + | +/− | ++ | ++ | − | − | +++ | − |
| Mi | OH in clay | 696 | − | − | − | − | − | − | + | + | − | − | − | + | − | − | − | − | + | + | − | + | ++ | − |
| S | S-O (b.) | 667 | + | + | − | + | + | +/− | +/− | − | + | − | + | +/− | +/− | + | +++ | +/− | +/− | − | + | ++ | +/− | − |
| S | S-O (b.) | 601 | + | + | + | + | + | +/− | +/− | ++ | + | +/− | + | +/− | − | − | +++ | − | +/− | +/− | +/− | + | +/− | − |
